# Prognostic Impact of MYC/TP63 Molecular Subtypes in Adenoid Cystic Carcinoma: A Meta-Analysis †

**DOI:** 10.3390/cancers18091426

**Published:** 2026-04-29

**Authors:** Karthik N. Rao, Prajwal Dange, M. P. Sreeram, Andrés Coca-Pelaz, Göran Stenman, Renata Ferrarotto, Teertha Shetty, Abbas Agaimy, Alfio Ferlito

**Affiliations:** 1 Department of Head and Neck Oncology, Sri Shankara Cancer Hospital and Research Center, Bangalore 560004, India; prajwal.dange@gmail.com (P.D.); drsreeram111@gmail.com (M.P.S.); drteerthaprajwal@gmail.com (T.S.); 2 Department of Otolaryngology, Hospital Universitario Central de Asturias, University of Oviedo, ISPA, IUOPA, CIBERONC, 33003 Oviedo, Spain; acocapelaz@yahoo.es; 3 Department of Pathology, Sahlgrenska Center for Cancer Research, Sahlgrenska University Hospital, University of Gothenburg, 40530 Gothenburg, Sweden; goran.stenman@gu.se; 4 Department of Thoracic/Head and Neck Medical Oncology, The University of Texas MD Anderson Cancer Center, Houston, TX 77030, USA; rferrarotto@mdanderson.org; 5 Institute of Pathology, Erlangen University Hospital, Friedrich Alexander University of Erlangen-Nuremberg, Krankenhausstrasse 8-10, 91054 Erlangen, Germany; abbas.agaimy@uk-erlangen.de; 6 Coordinator International Head and Neck Scientific Group, 35030 Padua, Italy; profalfioferlito@gmail.com

**Keywords:** adenoid cystic carcinoma, molecular subtyping, MYC, TP63, prognosis

## Abstract

**Background:** Adenoid cystic carcinoma (ACC) demonstrates marked clinical heterogeneity that is inadequately explained by conventional histopathologic and staging systems alone. Recent studies have identified two molecular subtypes based on transcriptomic profiling and MYC/TP63 expression (ACC I: MYC-high/TP63-low; ACC II: MYC-low/TP63-high) with potential prognostic significance. However, the magnitude and consistency of their survival impact remain uncertain. **Methods:** A systematic review and meta-analysis were conducted in accordance with PRISMA guidelines. PubMed, Embase, and PubMed Central were searched through January 2026 for studies reporting overall survival in ACC stratified by MYC/TP63 molecular subtype. Hazard ratios (HRs) were pooled using random-effects models. Heterogeneity, subgroup analyses by classification method, sensitivity analyses, cumulative meta-analysis, influence diagnostics, and publication bias assessment were performed. **Results:** Five independent cohorts from two publications comprising 247 patients (90 ACC I, 157 ACC II) were included. ACC I was associated with significantly worse overall survival compared with ACC II, with a pooled HR of 3.88 (95% CI: 2.55–5.90; *p* < 0.001). No statistical heterogeneity was observed (I^2^ = 0%). Prognostic separation was consistent across RNA sequencing and immunohistochemistry-based classification methods. **Conclusions:** Transcriptomic and MYC/TP63-based molecular subtyping provides strong and reproducible prognostic stratification in ACC. ACC I tumors confer an approximately four-fold higher mortality risk compared with ACC II tumors. Incorporation of molecular subtype into routine diagnostic and clinical decision-making may improve risk stratification, surveillance strategies, and future trial design in ACC.

## Simple Summary

Adenoid cystic carcinoma is a rare cancer of the salivary glands that behaves unpredictably, making it difficult for doctors to accurately estimate prognosis and plan follow-up. Traditional methods based on tumor appearance and stage do not fully explain why some patients experience poorer outcomes than others. Recent research suggests that differences at the molecular level may help divide this cancer into two biologically distinct types with different outcomes. In this study, we systematically reviewed available evidence and combined data from multiple patient groups to better understand how these molecular differences affect survival. Our findings show that one subtype is consistently associated with a higher risk of death. This information may help improve risk assessment, guide patient counseling, and support the development of more personalized treatment and follow-up strategies in the future.

## 1. Introduction

Adenoid cystic carcinoma (ACC) is a rare malignant epithelial tumor of salivary gland origin characterized by indolent growth, frequent perineural invasion, and a strong propensity for locoregional recurrence and late distant metastasis [1]. Despite relatively favorable short-term survival, long-term outcomes remain poor, with many patients experiencing disease progression years after initial treatment [2]. This unpredictable clinical course has long challenged clinicians, highlighting the limitations of conventional prognostic factors such as tumor site, stage, and histologic pattern.

Traditionally, ACC has been classified based on architectural growth patterns—cribriform, tubular, and solid—with the solid subtype being linked to worse outcomes [3]. However, histologic grading suffers from interobserver variability and fails to fully explain the marked heterogeneity in survival observed among patients with similar clinicopathologic features [4]. Likewise, staging systems alone, while useful for treatment planning, do not reliably predict long-term disease behavior [5]. These limitations have driven increasing interest in molecular classification systems capable of providing biologically meaningful and clinically actionable prognostic information.

Recent transcriptomic and proteomic studies have identified two major molecular subtypes of ACC based on MYC and TP63 expression patterns: ACC I (MYC-high, TP63-low) and ACC II (MYC-low, TP63-high) [6]. ACC I tumors also had significantly more copy number alterations, including 1p36 and PARK2 (6q26) deletions (6q), and reduced expression of TP73 (1p36) compared to ACC II tumors [7]. This classification reflects underlying molecular and biological differences in tumor differentiation, proliferation, and aggressiveness. ACC I tumors are characterized by enhanced proliferative signaling, loss of myoepithelial differentiation, and aggressive clinical behavior, whereas ACC II tumors retain features of myoepithelial lineage differentiation and demonstrate more favorable outcomes. Importantly, this molecular dichotomy can be determined using either RNA sequencing or immunohistochemistry (IHC), making it potentially feasible for routine clinical implementation.

Several independent cohort studies have reported significantly worse survival in patients with ACC I compared with ACC II tumors [6,7,8]. Given the relative rarity of ACC, most individual cohorts are underpowered to provide definitive estimates of effect size and the true magnitude and consistency of the prognostic impact of molecular subtyping. Furthermore, no prior meta-analysis has systematically synthesized the available evidence to quantify survival differences between these molecular subtypes.

Therefore, the present systematic review and meta-analysis was conducted to comprehensively evaluate the prognostic significance of MYC/TP63-based molecular subtyping in ACC. Specifically, we aimed to compare outcomes between ACC I and ACC II tumors across all eligible cohorts, assess heterogeneity of effect, evaluate robustness through sensitivity and influence analyses, and examine whether prognostic performance differs according to molecular classification methodology. By synthesizing all available high-quality evidence, this study seeks to establish this molecular subtype as a clinically meaningful prognostic biomarker in ACC.

## 2. Materials and Methods

### 2.1. Study Design and Reporting Guidelines

This systematic review and meta-analysis was conducted and reported in accordance with the Preferred Reporting Items for Systematic Reviews and Meta-Analyses (PRISMA) statement guidelines [9]. The methodological quality of included studies was assessed using the Newcastle–Ottawa Scale (NOS) for observational studies [10]. This systematic review was not prospectively registered in a public database. The PRISMA 2020 checklist is provided in the Supplementary Materials (Figure S6).

### 2.2. Literature Search Strategy

A comprehensive literature search was performed across multiple electronic databases including PubMed, Embase, PubMed Central, and ClinicalTrials.gov to identify all relevant studies reporting survival outcomes in adenoid cystic carcinoma stratified by molecular subtypes as proposed by Ferrarotto et al. [6]. The search encompassed all available literature from database inception through January 2026 without language restrictions initially, though only English-language studies were ultimately included in the final analysis.

The search strategy employed a combination of medical subject headings and free-text terms related to the condition, molecular classification, and clinical outcomes. The primary search terms included “adenoid cystic carcinoma” combined with terms related to molecular classification including “MYC,” “TP63,” “molecular subtype,” “transcriptomic classification,” and “gene expression.” These were further combined with outcome-related terms including “survival,” “prognosis,” “mortality,” and “outcome.” The complete search strategy was adapted appropriately for each database to account for variations in controlled vocabulary and search syntax.

Reference lists of all included studies and relevant review articles were manually screened to identify additional studies that may have been missed by the electronic search strategy. Citation tracking was performed to identify studies that cited included studies.

### 2.3. Study Selection and Eligibility Criteria

Two independent reviewers (PSD and KNR) conducted the study selection process in two stages. Initially, titles and abstracts of all retrieved citations were screened against predetermined eligibility criteria to identify potentially relevant studies. Subsequently, full-text articles of all potentially eligible studies were obtained and assessed for final inclusion.

Studies were considered eligible for inclusion if they reported survival outcomes in patients with adenoid cystic carcinoma stratified by molecular subtypes based on MYC and TP63 gene expression patterns as proposed by Ferrarotto et al. [6]. Eligible studies needed to classify tumors as ACC I (MYC-high, TP63-low) versus ACC II (MYC-low, TP63-high) using either RNA sequencing or IHC. Studies were required to report hazard ratios for overall survival with corresponding confidence intervals, or provide sufficient data to calculate these effect estimates. A minimum of 10 patients per molecular subtype was required to ensure stability of survival estimates and to minimize sparse-data bias associated with very small subgroup analyses. Very small samples may produce unstable effect estimates and inflated variance, thereby reducing the reliability of pooled results [11,12]. While the primary analysis applied a predefined inclusion criterion of ≥10 patients per molecular subtype to ensure stability of effect estimates, a supplementary sensitivity analysis was performed including all eligible studies irrespective of sample size. The results of this expanded analysis are presented in the Supplementary Materials.

Studies were excluded if they classified adenoid cystic carcinoma based solely on histologic grading, MYB or MYBL1 fusion status alone, epithelial versus myoepithelial predominance, or microRNA expression profiles without corresponding MYC/TP63 classification. Studies reporting only on preclinical models, case reports with fewer than 10 patients per subtype, or studies that did not report overall survival outcomes were also excluded. When multiple publications reported on overlapping patient cohorts, the most recent or most comprehensive publication was included to avoid duplicate counting of patients.

### 2.4. Data Extraction

Study-level data extracted included publication details such as first author name, year of publication, country of origin, and study institution. Methodological characteristics including study design, sample size, patient enrollment period, and length of follow-up were systematically recorded. Details regarding the molecular classification method employed, including specific assay platforms for RNA sequencing studies or antibody clones and scoring systems for IHC-based studies, were documented.

Patient-level data extracted included demographic characteristics such as age distribution and gender proportions, tumor characteristics including primary tumor site, histologic grade when reported, and TNM staging information when available. For each molecular subtype, the number of patients classified as ACC I and ACC II was recorded along with corresponding survival outcomes.

Survival data were extracted as hazard ratios with 95% confidence intervals for overall survival comparing ACC I versus ACC II tumors. When hazard ratios were not directly reported in the text, they were extracted from tables or calculated from published Kaplan–Meier survival curves using established digitization methods. Median survival times for each molecular subtype were recorded when reported. The primary outcome of interest was overall survival, defined as time from diagnosis or treatment initiation to death from any cause.

### 2.5. Quality Assessment

#### 2.5.1. Level of Evidence

The level of evidence in eligible studies was independently assessed by two reviewers (PSD and KNR) using the Oxford Centre for Evidence-Based Medicine levels of evidence framework.

#### 2.5.2. Methodological Quality

The methodological quality of included studies was systematically assessed using the NOS for cohort studies [10,13]. The NOS evaluates studies across three domains: selection of study groups (4 stars), comparability of groups (2 stars), and assessment of outcomes (3 stars), with a maximum total score of 9 stars. Studies scoring 7 or more stars were classified as high quality, those scoring 4–6 stars as medium quality, and those scoring less than 4 stars as low quality [13].

Individual study quality scores were tabulated, and studies assessed as having high risk of bias were considered for exclusion in sensitivity analyses to assess the pooled effect estimates to study quality.

### 2.6. Statistical Analysis

#### 2.6.1. Primary Analysis

The primary analysis involved pooling hazard ratios for overall survival across included studies using meta-analytic methodology. The pooled hazard ratio represents a weighted average of individual study estimates, with more precise studies (those with smaller standard errors) contributing proportionally greater weight. The random-effects model was considered the primary analysis as it assumes that true effect sizes may vary across studies due to differences in patient populations, molecular classification methods, and clinical settings. The DerSimonian–Laird method was used to estimate between-study variance (tau-squared, τ^2^) in the random-effects model [14]. The fixed-effect model, which assumes a single true effect size across all studies, was also calculated for comparison. Pooled hazard ratios with corresponding 95% confidence intervals were calculated, and statistical significance was assessed using two-tailed z-tests with alpha set at 0.05.

#### 2.6.2. Heterogeneity Assessment

Statistical heterogeneity among studies was quantitatively assessed using three complementary measures. The I^2^ statistic was calculated to determine the percentage of total variation across studies attributable to heterogeneity rather than chance, with values of 0–40%, 40–75%, and 75–100% interpreted as representing low, moderate, and high heterogeneity, respectively [15]. The between-study variance (tau-squared, τ^2^) was estimated using the DerSimonian–Laird estimator to quantify the absolute magnitude of heterogeneity [16]. Cochran’s Q statistic was calculated to test the null hypothesis of homogeneity, with statistical significance set at *p* < 0.10 given the low statistical power of this test when the number of studies is small [17].

#### 2.6.3. Effect Size Visualization

Forest plots were constructed to provide visual representation of individual study effect estimates alongside the pooled effect size with confidence intervals [18]. Studies were ordered chronologically by publication year. The size of each study marker was proportional to the inverse of the variance, reflecting the relative weight of each study in the meta-analysis. Both fixed-effect and random-effects pooled estimates were displayed. The prediction interval, representing the range in which the true effect size in a future similar study would be expected to fall with 95% probability, was also calculated and displayed.

#### 2.6.4. Sensitivity and Subgroup Analyses

Leave-one-out sensitivity analysis was performed by systematically excluding each study in turn and recalculating the pooled effect estimate and heterogeneity statistics [19]. This approach identifies studies with disproportionate influence on the overall pooled estimate and assesses the robustness of findings to individual study inclusion.

Subgroup meta-analysis was conducted. Studies were stratified into those using RNA sequencing versus IHC for MYC and TP63 classification. Subgroup-specific pooled hazard ratios were calculated, and formal statistical testing for differences between subgroups was performed using meta-regression with a test for subgroup differences. Cumulative meta-analysis was performed with studies ordered chronologically to examine the evolution of evidence over time and to assess whether the pooled effect estimate became more precise and stable as evidence accumulated [20].

#### 2.6.5. Influential Study Assessment (Exploratory)

As an exploratory analysis given the small number of studies (k = 5), influence diagnostics were calculated to identify studies that exerted disproportionate influence on the meta-analysis results. DFBETAS statistics were computed to quantify the change in the pooled estimate resulting from exclusion of each study, standardized by the standard error. Cook’s distance was calculated to provide an overall measure of each study’s influence on the fitted model [21]. Hat values (leverage statistics) were computed to identify studies with unusual covariate patterns that might exert high leverage on the pooled estimate. Externally studentized residuals were calculated to identify studies with effect sizes that deviated substantially from the pooled estimate.

#### 2.6.6. Publication Bias Evaluation (Exploratory)

Assessment of publication bias was limited by the small number of included studies (n = 5). Funnel plot analysis was not performed, as fewer than 10 studies were available, rendering visual assessment of asymmetry unreliable. Formal statistical tests for funnel plot asymmetry, including Egger’s regression test and Begg’s rank correlation test, were not performed due to insufficient statistical power with fewer than 10 studies [22].

#### 2.6.7. Statistical Software

All analyses were conducted using R statistical software version 4.5.1 (R Foundation for Statistical Computing, Vienna, Austria). Meta-analyses were performed in R using the ‘meta’ and ‘metafor’ packages [23,24]. Statistical significance was set at *p* < 0.05 for all analyses except heterogeneity assessment, where *p* < 0.10 was used given the low power of the Q-test with small numbers of studies. All reported *p*-values are two-sided.

## 3. Results

### 3.1. Literature Retrieval and Study Selection

**Figure 1:**
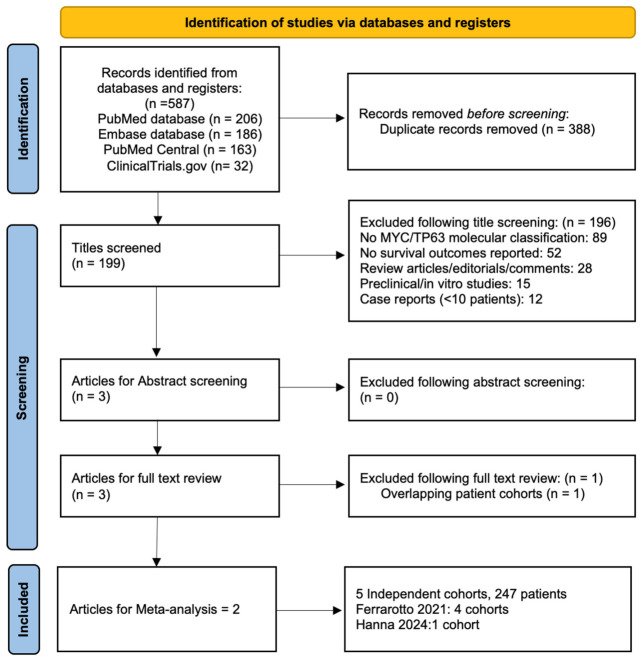
PRISMA Flow Diagram.

The initial literature search identified 587 records across three databases and one clinical trial registry. PubMed yielded 206 articles, Embase contributed 186 articles, PubMed Central provided 163 articles, and ClinicalTrials.gov contributed 32 records. After removal of 388 duplicates, 199 unique citations remained for title screening. Following title screening, 196 articles were excluded for the following reasons: 89 did not classify tumors based on MYC/TP63 expression; 52 did not report survival outcomes; 28 were review articles, editorials, or comments; 15 were preclinical or in vitro studies; and 12 were case reports with fewer than 10 patients. Three articles proceeded to abstract screening, with no exclusions at this stage. All 3 articles underwent full-text evaluation. After applying the predetermined inclusion and exclusion criteria, 1 article was excluded due to overlapping patient cohorts with a subsequently published study. Ultimately, 2 primary publications reporting on 5 independent cohorts comprising 247 patients (90 ACC I, 157 ACC II) met all inclusion criteria and were included in the quantitative synthesis (Figure 1).

### 3.2. Study Characteristics and Quality Assessment

The meta-analysis comprised 5 independent cohorts reported across 2 primary publications [6,8] spanning the period from 2021 to 2024. The included studies encompassed 247 patients with adenoid cystic carcinoma classified by molecular subtype, including 90 ACC I tumors (36.4%) and 157 ACC II tumors (63.6%). These studies originated from research groups in the United States and represented multi-institutional collaborative efforts incorporating both retrospective cohort analyses and prospectively collected biobanked specimens (Table 1).

The landmark study by Ferrarotto et al. (2021) contributed four independent validation cohorts to this meta-analysis [6]. The Discovery cohort (n = 54) employed RNA sequencing for molecular classification and provided validation of the MYC/TP63-based classification system. Three additional validation cohorts utilized different methodological approaches: The Bell cohort (n = 36) applied RNA sequencing to an independent patient series, the Frerich cohort (n = 37) similarly employed RNA sequencing methodology, and the IHC cohort (n = 58) demonstrated the feasibility of classification using IHC for MYC and TP63 proteins. All four cohorts in Ferrarotto et al. were independent patient populations without overlap, representing diverse institutional settings and patient populations [6].

The study by Hanna et al. (2024) analyzed a large multi-institutional cohort (n = 62 with complete molecular and survival data) from the Caris Life Sciences molecular profiling database [8]. This cohort employed comprehensive transcriptomic/genomic profiling including RNA sequencing to classify tumors according to MYC and TP63 expression patterns. The Hanna et al. cohort represented the largest single cohort in the meta-analysis and provided important validation of the prognostic significance of molecular subtyping in a real-world clinical setting [8].

Molecular classification methods varied across cohorts, with four cohorts employing RNA sequencing and one utilizing IHC. The RNA sequencing-based cohorts used validated gene expression signatures to classify tumors as ACC I or ACC II. The IHC-based cohort employed standardized antibodies against MYC and TP63 proteins with predefined scoring criteria to assign molecular subtype classification. Despite methodological heterogeneity in classification approaches, all studies employed the fundamental dichotomy of MYC-high/TP63-low (ACC I) versus MYC-low/TP63-high (ACC II) classification. Scoring thresholds followed the corrected criteria reported in the published erratum [6].

Quality assessment using the NOS revealed uniformly high methodological quality across all included studies. The Ferrarotto et al. [6] cohorts (Discovery, Bell, Frerich, IHC) each received scores of 8 stars. The Hanna et al. [8] cohort similarly achieved a score of 8 stars. Across all cohorts, the single deducted star was in the Comparability domain, reflecting the absence of multivariable adjustment for potential confounders—a limitation inherent to the available data and acknowledged in our Limitations section.

Patient demographics across cohorts reflected typical adenoid cystic carcinoma populations with slight female predominance and median age in the sixth decade. Tumor characteristics varied across studies, encompassing adenoid cystic carcinomas arising from major and minor salivary glands as well as other sites. Stage distribution included both early and advanced disease, though specific TNM staging information varied in completeness across studies. Follow-up duration was generally adequate for survival analysis, with median follow-up periods ranging from 3 to 10 years across cohorts.

### 3.3. Primary Outcome: Overall Survival

The primary analysis pooled hazard ratios for overall survival comparing ACC I versus ACC II across all five cohorts. ACC I tumors demonstrated significantly worse overall survival compared to ACC II tumors, with a pooled hazard ratio of 3.88 (95% confidence interval: 2.55 to 5.90, *p* < 0.001) using the random-effects model. This indicates that patients with ACC I had a nearly four-fold higher risk of death compared to those with ACC II tumors.

Individual study hazard ratios ranged from 3.04 (Caris cohort) to 6.00 (Bell cohort), with all five cohorts demonstrating consistent direction of effect favoring better survival in ACC II tumors. The 95% prediction interval, which estimates where the true effect in a future similar study would likely fall, ranged from 2.14 to 7.03, indicating substantial effect size even when accounting for potential heterogeneity in future studies.

**Figure 2:**
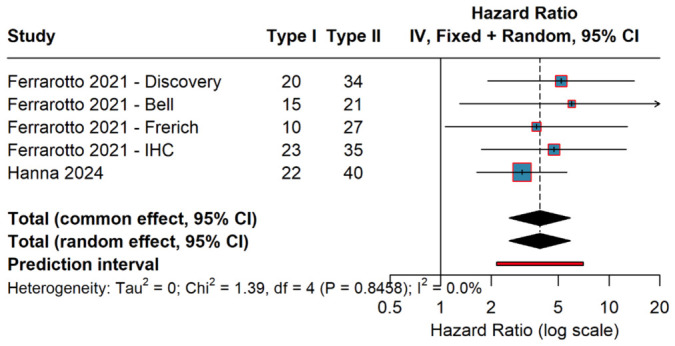
Forest Plot of Overall Survival—Main Analysis. Cohorts from Ferrarotto et al. (2021) [6] and Hanna et al. (2024) [8].

The forest plot (Figure 2) demonstrates remarkable consistency of effect across all cohorts, with narrow confidence intervals for most studies reflecting adequate sample sizes and precision of effect estimates. All individual study confidence intervals excluded the null value of hazard ratio = 1.0, indicating statistically significant associations in each cohort independently. The visual representation clearly illustrates the concordance of findings across different patient populations, institutions, and molecular classification methodologies.

### 3.4. Heterogeneity Assessment

Statistical assessment of heterogeneity revealed homogeneity of effect sizes across the five cohorts. The I^2^ statistic was 0% (95% CI: 0% to 68%), indicating that essentially none of the observed variance in effect sizes could be attributed to true heterogeneity between studies. The estimated between-study variance (tau-squared, τ^2^) was 0. Cochran’s Q test for heterogeneity yielded χ^2^ = 1.39 (degrees of freedom = 4, *p* = 0.85), providing no evidence against the null hypothesis of homogeneity.

### 3.5. Subgroup Analysis by Classification Method

Subgroup meta-analysis was performed to examine whether the prognostic value of molecular classification differed according to the methodology employed for subtype determination. Four cohorts utilized RNA sequencing for molecular classification (Discovery, Bell, Frerich, and Hanna cohorts, total n = 189), while one cohort employed IHC (IHC cohort, n = 58).

Among RNA sequencing-based cohorts, the pooled hazard ratio was 3.72 (95% CI: 2.34 to 5.91, *p* < 0.001), with no evidence of heterogeneity (I^2^ = 0%, τ^2^ = 0, *p* = 0.75). The single IHC-based cohort reported a hazard ratio of 4.70 (95% CI: 1.75 to 12.64). Formal testing for subgroup differences revealed no statistically significant heterogeneity between classification methods (χ^2^ = 0.17, degrees of freedom = 1, *p* = 0.68).

**Figure 3:**
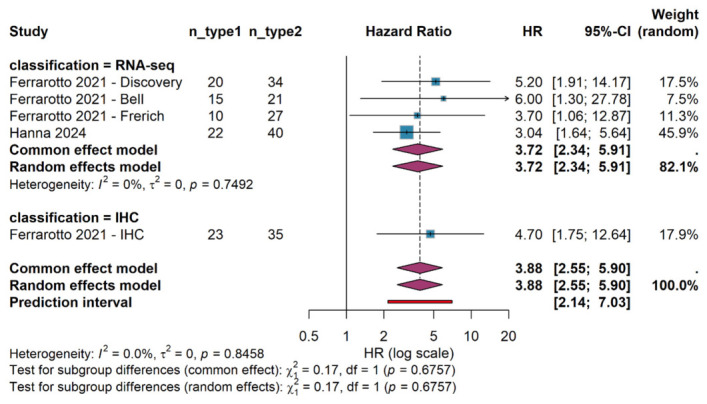
Forest Plot of Subgroup Analysis by Classification Method. Cohorts from Ferrarotto et al. (2021) [6] and Hanna et al. (2024) [8].

These findings demonstrate that the prognostic impact of molecular subtype classification is preserved regardless of whether classification is performed using RNA sequencing or IHC. This has important practical implications, as IHC-based classification is more readily implemented in routine clinical practice compared to RNA sequencing as illustrated in the subgroup forest plot (Figure 3).

### 3.6. Sensitivity Analysis

In this analysis, the meta-analysis was recalculated five times, each time omitting one study. When the Discovery cohort was excluded, the pooled hazard ratio was 3.65 (95% CI: 2.30 to 5.79). Exclusion of the Bell cohort yielded a pooled HR of 3.75 (95% CI: 2.42 to 5.79). Omitting the Frerich cohort resulted in a pooled HR of 3.91 (95% CI: 2.50 to 6.09). Excluding the IHC cohort produced a pooled HR of 3.72 (95% CI: 2.34 to 5.91). Finally, omission of the Hanna cohort, which contributed the largest sample size, resulted in the highest pooled HR of 4.78 (95% CI: 2.70 to 8.44).

**Figure 4:**
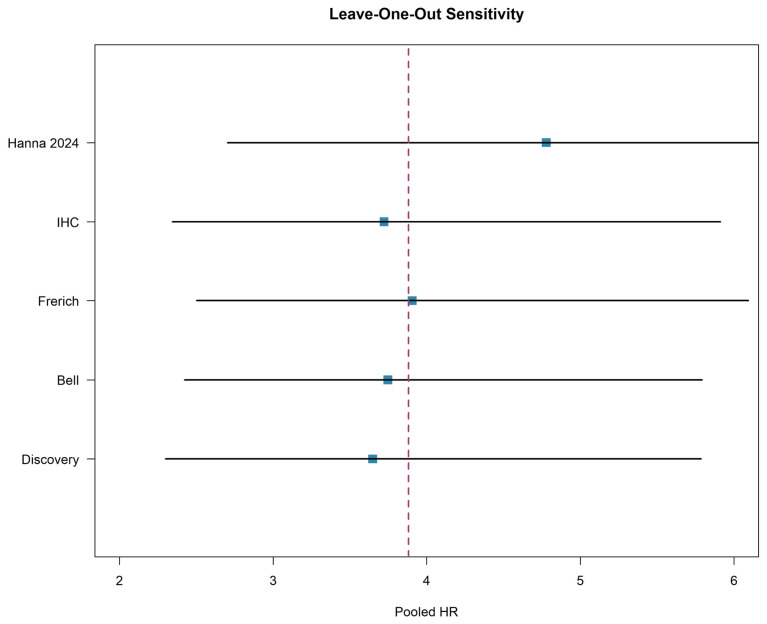
Leave-One-Out Sensitivity Analysis. Cohorts from Ferrarotto et al. (2021) [6] and Hanna et al. (2024) [8]. Squares represent pooled estimates when each cohort is omitted; the dashed vertical line represents the overall pooled estimate.

Across all leave-one-out iterations, the pooled hazard ratio remained statistically significant (all *p* < 0.001) and ranged narrowly from 3.65 to 4.78. The 95% confidence intervals for all iterations excluded the null value. The slightly higher pooled estimate when the Hanna cohort is excluded reflects the relatively conservative effect size in that study (HR = 3.04), though the overall conclusion remains unchanged. as shown in the leave-one-out sensitivity plot (Figure 4).

### 3.7. Cumulative Meta-Analysis

Studies from the Ferrarotto et al. [6] publication (2021) were added first in the sequence reported (Bell, Frerich, Discovery, IHC), followed by the Hanna et al. [8] study (2024).

With the addition of the first study (Bell cohort), the pooled HR was 6.00 (95% CI: 1.30 to 27.78), showing a strong but imprecise effect estimate. Addition of the Frerich cohort narrowed the confidence interval considerably, yielding a pooled HR of 4.48 (95% CI: 1.71 to 11.79). With the inclusion of the Discovery cohort as the third study, the pooled HR was 4.82 (95% CI: 2.40 to 9.66). The addition of the IHC cohort further refined the estimate to HR = 4.78 (95% CI: 2.70 to 8.44). Finally, incorporation of the Hanna cohort brought the pooled estimate to its final value of HR = 3.88 (95% CI: 2.55 to 5.90).

**Figure 5:**
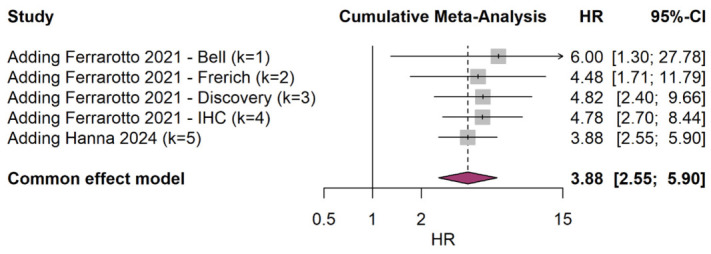
Cumulative Meta-Analysis. Cohorts from Ferrarotto et al. (2021) [6] and Hanna et al. (2024) [8].

The cumulative analysis demonstrates that the effect size estimate was stabilized by the third study, with progressive narrowing of confidence intervals as additional studies contributed data. The hazard ratio remained consistently elevated (>3.5) throughout the cumulative process, and statistical significance was achieved by the second study and maintained thereafter as shown in the cumulative meta-analysis plot (Figure 5).

### 3.8. Influence Diagnostics

DFBETAS statistics, which quantify the standardized change in the pooled estimate when each study is excluded, revealed that the Hanna cohort had the largest influence (DFBETAS = −0.98), followed by the Discovery cohort (DFBETAS = 0.35) and IHC cohort (DFBETAS = 0.29). The Bell and Frerich cohorts showed minimal influence (DFBETAS = 0.23 and −0.03, respectively). Using the conventional threshold of |DFBETAS| > 2/√k = 0.89 for k = 5 studies, the Hanna cohort slightly exceeded the conventional threshold (|DFBETAS| = 0.98 > 0.89), although Cook’s distance remained < 1.0 and residual diagnostics showed no outlier behavior.

Cook’s distance, providing an overall measure of study influence, was highest for the Hanna cohort (0.95) but remained below the conventional threshold of 1.0 that would indicate excessive influence. Other studies showed substantially lower Cook’s distances (Discovery = 0.10, Bell = 0.05, Frerich = 0.00, IHC = 0.04), confirming minimal individual study influence on the overall model.

Hat values (leverage statistics) indicated that the Hanna cohort had the highest leverage (0.47) due to its larger sample size and precise effect estimate, while other studies showed more moderate leverage values (Discovery = 0.17, Bell = 0.08, Frerich = 0.12, IHC = 0.18). Despite its high leverage, the Hanna study’s effect estimate aligned well with other studies, resulting in low residual deviation.

Externally studentized residuals revealed that all studies fell well within the expected range, with values between −1.1 and 0.6 standard deviations from the pooled estimate. No study exhibited residuals exceeding ±2 standard deviations, indicating an absence of outliers. The Hanna cohort showed a negative residual (−1.05), reflecting its slightly more conservative effect estimate, while other studies showed smaller positive or near-zero residuals.

**Figure 6:**
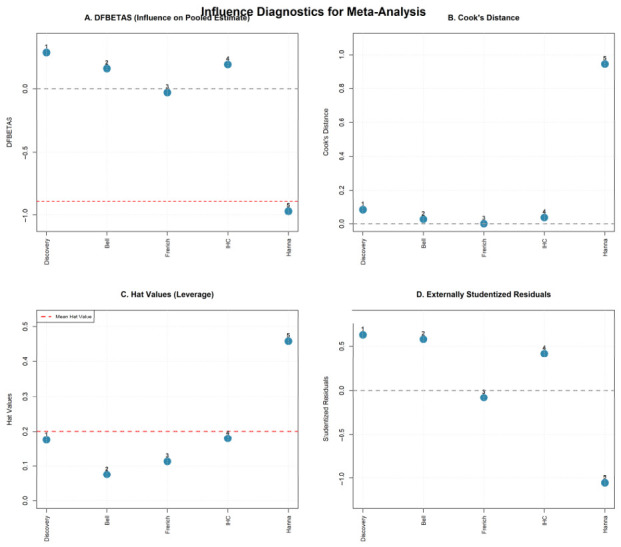
Influence Diagnostics—Four-panel plot showing DFBETAS, Cook’s Distance, Hat Values, and Studentized Residuals. In subfigure (**A**) (DFBETAS), numbers indicate DFBETAS values per cohort; red dashed lines represent the influence threshold (±2/√k = ±0.89). The grey dashed line in subfigures (**A**,**B**,**D**) represents the null effect (HR = 1.0). Cohorts from Ferrarotto et al. (2021) [6] and Hanna et al. (2024) [8].

While the Hanna cohort contributes substantial weight due to its larger sample size, its effect estimate is concordant with other studies, and its influence remains within acceptable bounds. These results are summarised in the influence diagnostics panel (Figure 6).

### 3.9. Publication Bias Assessment (Exploratory)

Formal assessment of publication bias was not feasible due to the small number of included studies (n = 5). With fewer than 10 studies, funnel plot analysis is unreliable and was therefore not performed.

Egger’s regression test and Begg’s rank correlation test were not performed due to insufficient statistical power with fewer than 10 studies.

Nevertheless, the possibility of publication bias cannot be definitively excluded given the small number of studies and the inability to conduct formal statistical tests. Future updates of this meta-analysis incorporating additional studies will allow more definitive assessment of potential publication bias.

A comprehensive summary of all meta-analysis results, including primary outcomes, heterogeneity assessment, subgroup analyses, sensitivity analyses, influence diagnostics, and publication bias evaluation, is presented in Table 2.

### 3.10. Supplementary Sensitivity Analysis

A pre-planned supplementary sensitivity analysis was conducted incorporating the Economopoulou et al. (2025) [25] cohort (ACC I n = 8, ACC II n = 39, HR = 3.67, 95% CI: 1.44–9.38), which was excluded from the primary analysis because the ACC I subgroup contained fewer than 10 patients. Including this sixth cohort yielded a pooled HR of 3.84 (95% CI: 2.62–5.62) with I^2^ = 0%, virtually identical to the primary analysis (HR = 3.88). The prediction interval for the 6-cohort analysis was 2.33 to 6.32. The 6-cohort sensitivity analysis is fully presented in the Supplementary Materials, including the forest plot (Supplementary Figure S1), subgroup analysis by classification method (Supplementary Figure S2), leave-one-out sensitivity analysis (Supplementary Figure S3), cumulative meta-analysis (Supplementary Figure S4), and influence diagnostics (Supplementary Figure S5). These results confirm that the prognostic effect of MYC/TP63 subtyping is robust and not dependent on the inclusion threshold or a single research group.

## 4. Discussion

This systematic review and meta-analysis demonstrates that molecular subtyping of ACC based on MYC and TP63 expression provides powerful and consistent prognostic discrimination. Across five independent cohorts comprising 247 patients, ACC I patients were associated with a nearly four-fold higher risk of death compared with ACC II tumors [6,8]. Remarkably, this effect was highly consistent across all cohorts, with no detectable statistical heterogeneity, and remained robust across multiple sensitivity and influence analyses.

The pooled hazard ratio of 3.88 indicates that the molecular subtype is among the strongest prognostic factors currently identified in ACC. This magnitude of effect exceeds that reported for many traditional clinico-pathologic variables, including histologic pattern and, in some series, even tumor stage. Importantly, all individual cohorts demonstrated statistically significant survival differences in the same direction. The prediction interval suggests that even in future independent studies, ACC I tumors are expected to confer at least a two-fold increased mortality risk, reinforcing the generalizability of the findings.

The transcriptomic study by Romani et al. analyzed 46 ACC tumors and reported that low TP63 expression (ACC I) was associated with significantly worse DFS (HR 0.34, 95% CI 0.15–0.77, *p* = 0.01). This correlated with high-grade disease, with p63 negativity observed in 82% of grade 3 ACC tumors compared with universal positivity in grade 1–2 tumors. High MYC expression showed a trend toward worse survival (HR 2.60, 95% CI 0.94–7.15, *p* = 0.065). TP63-negative tumors demonstrated increased MYC expression (fold change 1.31, *p* = 0.008) with enrichment of MYC targets and cell-cycle pathways. These biological characteristics are consistent with the aggressive phenotype corresponding to ACC I compared with ACC II [26]. However, the study could not be incorporated into the quantitative meta-analysis as overall survival outcomes were not reported.

A multicenter retrospective study from Greece including 47 patients classified ACC into ACC-I (n = 8) and ACC-II (n = 39) and reported significantly improved survival in ACC-II, with median overall survival of 135 months versus 50 months in ACC-I (*p* = 0.0066) [25]. As the study did not report hazard ratios, we estimated the HR using the Tierney method [27] from their survival data, yielding an HR of 3.67 (95% CI 1.44–9.38) favoring the ACC II subtype. It should be noted that the indirectly calculated HR demonstrated wide variance due to the small sample size. However, the study was excluded from the present meta-analysis because the ACC I subgroup contained fewer than 10 patients, which did not meet our predefined inclusion criteria. The observed survival trend was consistent with the direction of effect seen in our meta-analysis.

From a clinical standpoint, these results support molecular subtype as a reliable tool for risk stratification at diagnosis. Patients with ACC I tumors represent a high-risk population who may benefit from intensified surveillance, early detection of metastasis, and consideration for clinical trials evaluating novel systemic therapies.

The observed prognostic divergence is biologically plausible. MYC overexpression is a well-established oncogenic driver of cellular proliferation, genomic instability, and tumor aggressiveness across multiple malignancies [28]. Conversely, TP63 is associated with myoepithelial differentiation and maintenance of epithelial integrity [29]. Loss of TP63 expression reflects dedifferentiation and acquisition of a more aggressive tumor phenotype [30].

ACC I tumors, characterized by MYC-high and TP63-low expression, represent a biologically aggressive entity with lower differentiation and increased metastatic potential. In contrast, ACC II tumors retain myoepithelial features and exhibit a more indolent clinical course. The consistent prognostic separation observed across cohorts strongly suggests the concept that ACC is a spectrum of biologically distinct subtypes.

An important strength of this meta-analysis is the demonstration that prognostic discrimination is preserved across both RNA sequencing and IHC-based classification methods. The absence of significant subgroup differences indicates that assessment of MYC and TP63 protein levels using IHC can reliably reproduce transcriptomic prognostic stratification.

This finding has major practical implications. While RNA sequencing may not be universally available, IHC is widely accessible, cost-effective, and easily integrated into routine pathology workflows. Thus, molecular subtyping can realistically be implemented in standard diagnostic practice without the need for advanced molecular infrastructure.

The clinical implications of these findings are important. Routine incorporation of molecular subtype into ACC reporting can enable risk-adapted surveillance, with ACC I patients requiring closer long-term monitoring for distant metastases. Prognostic counseling at diagnosis can be more accurate, and molecular subtype may assist in identifying candidates for systemic therapy trials. Future clinical trials should incorporate molecular stratification to avoid biological heterogeneity. This meta-analysis provides the first quantitative confirmation of the prognostic value of MYC/TP63 subtyping. These results align with emerging genomic data on aggressive ACC phenotypes [7] and position molecular subtype as a unifying, clinically meaningful classification framework.

### 4.1. Limitations

Although all available data were pooled, the number of eligible studies remains limited, restricting formal statistical assessment of publication bias. Most cohorts were retrospective, and residual confounding cannot be excluded despite high methodological quality. Importantly, all reported hazard ratios were derived from univariable (unadjusted) analyses, and whether MYC/TP63 subtype retains independent prognostic value after multivariable adjustment for established clinical variables remains unproven. Inconsistent reporting of stage, treatment, and tumor site precluded detailed subgroup analyses. The analysis was confined to overall survival, as data on disease-free survival, metastasis-free survival, cause-specific survival, and quality-of-life outcomes were scarce or not reported. Given the prolonged natural history of ACC, cause-specific survival would be particularly informative and should be prioritized in future studies. Finally, while molecular classification proved robust, standardized IHC scoring thresholds and formal inter-observer reproducibility studies are essential before routine clinical implementation.

### 4.2. Future Directions

Future research should prospectively validate molecular subtyping in large multicenter cohorts and integrate subtype with staging and radiologic parameters to develop composite prognostic models. Subtype-specific therapeutic vulnerabilities, particularly in MYC-driven tumors, warrant further investigation. The predictive value of molecular subtype for response to targeted and immunotherapeutic agents should also be explored. In parallel, standardized pathology reporting guidelines for MYC/TP63 IHC must be established to facilitate uniform clinical adoption. Ultimately, molecular subtyping has the potential to evolve beyond a prognostic marker and serve as a cornerstone for precision oncology in ACC. However, this requires that the molecular underpinnings of the differences between ACC I and ACC II tumors are resolved.

## 5. Conclusions

This meta-analysis establishes MYC/TP63-based subtyping as a powerful, consistent, and clinically meaningful prognostic biomarker in ACC. ACC I tumors carry an approximately four-fold higher risk of mortality compared with ACC II tumors, with remarkable reproducibility across cohorts and classification platforms. These findings provide strong preliminary evidence supporting further evaluation of molecular subtype into routine diagnostic evaluation, risk stratification, and future clinical trial design in ACC.

Declaration: This manuscript abstract was presented by TS at the 24th meeting on “Evidence-Based Management of Cancers in India (EBM 2026)” held at Tata Memorial Hospital, Mumbai, India.

## Figures and Tables

**Table 1:** Study Characteristics and Quality Assessment.

Study (Author, Year)	Cohort Name	Country	Study Design	Classification Method	Sample Size (ACC I/ACC II)	Hazard Ratio (95% CI)	Median Follow-Up	NOS Score
Ferrarotto et al., 2021 [6]	Discovery	USA	Retrospective	RNA-seq	20/34	4.40 (1.90–10.30)	13.0 years	8
Ferrarotto et al., 2021 [6]	Bell	USA	Retrospective	RNA-seq	15/21	6.00 (1.30–27.78)	NR	8
Ferrarotto et al., 2021 [6]	Frerich	USA	Retrospective	RNA-seq	10/27	3.70 (1.10–12.20)	NR	8
Ferrarotto et al., 2021 [6]	IHC	USA	Retrospective	IHC	23/35	4.70 (1.75–12.64)	NR	8
Hanna et al., 2024 [8]	Caris	USA	Retrospective	RNA-seq	22/40	3.04 (1.65–5.60)	NR	8
Total	5 cohorts	-	-	4 RNA-seq 1 IHC	90/157 (247 total)	3.88 (2.55–5.90) *	-	

NOS = Newcastle-Ottawa Scale; IHC = Immunohistochemistry; RNA-seq = RNA sequencing; NR = Not reported; USA = United States of America, * Pooled hazard ratio from random-effects meta-analysis.

**Table 2:** Comprehensive Summary of Meta-Analysis Results.

Analysis	Result	Interpretation
PRIMARY OUTCOME		
Overall Survival (ACC I vs. ACC II)	HR = 3.88 (95% CI: 2.55–5.90), *p* < 0.001	ACC I has ~4-fold higher risk of death
95% Prediction Interval	2.14 to 7.03	Future studies expected to show HR in this range
HETEROGENEITY ASSESSMENT		
I^2^ statistic	0% (95% CI: 0–68%)	No heterogeneity detected
Tau-squared (τ^2^)	0	No between-study variance
Cochran’s Q test	χ^2^ = 1.39, df = 4, *p* = 0.85	Homogeneous effect sizes across studies
SUBGROUP ANALYSIS BY CLASSIFICATION METHOD		
RNA-seq cohorts (n = 4, 189 patients)	HR = 3.72 (95% CI: 2.34–5.91), *p* < 0.001	Strong prognostic effect with RNA-seq
IHC cohort (n = 1, 58 patients)	HR = 4.70 (95% CI: 1.75–12.64)	Similar effect with IHC classification
Test for subgroup differences	χ^2^ = 0.17, df = 1, *p* = 0.68	No significant difference between methods
LEAVE-ONE-OUT SENSITIVITY ANALYSIS		
Excluding Discovery cohort	HR = 3.65 (95% CI: 2.30–5.79)	Result stable
Excluding Bell cohort	HR = 3.75 (95% CI: 2.42–5.79)	Result stable
Excluding Frerich cohort	HR = 3.91 (95% CI: 2.50–6.09)	Result stable
Excluding IHC cohort	HR = 3.72 (95% CI: 2.34–5.91)	Result stable
Excluding Hanna cohort	HR = 4.78 (95% CI: 2.70–8.44)	Result stable (slightly higher without largest study)
INFLUENCE DIAGNOSTICS		
DFBETAS (threshold: |0.89|)	Range: −0.98 to 0.35	Hanna: |DFBETAS| = 0.98 (>0.89); Cook’s distance <1 and residual diagnostics unremarkable.
Cook’s Distance (threshold: 1.0)	Range: 0.00 to 0.95	No study exceeds threshold
Studentized Residuals	Range: −1.1 to 0.6	No outliers detected (all within ±2 SD)
PUBLICATION BIAS		
Funnel plot analysis	Not performed (n < 10 studies)	Visual assessment unreliable with <10 studies
Formal statistical tests	Not performed (n < 10 studies)	Insufficient power for Egger’s/Begg’s tests

HR = Hazard Ratio; CI = Confidence Interval; df = degrees of freedom; SD = Standard Deviation; RNA-seq = RNA sequencing; IHC = Immunohistochemistry.

## Data Availability

No new data were created or analyzed in this study.

## Supplementary Materials

The following supporting information can be downloaded at: https://www.mdpi.com/article/10.3390/cancers18091426/s1, Figure S1: Forest Plot—6-Cohort Sensitivity Analysis; Figure S2: Subgroup Analysis by Classification Method—6-Cohort Analysis; Figure S3: Leave-One-Out Sensitivity Analysis—6-Cohort Analysis; Figure S4: Cumulative Meta-Analysis—6-Cohort Analysis; Figure S5: Influence Diagnostics—6-Cohort Analysis (Exploratory). Figure S6: The PRISMA 2020 checklist.
