# Supplementary material for: Prognostic Impact of MYC/TP63 Molecular Subtypes in Adenoid Cystic Carcinoma: A Meta-Analysis
Source: Cancers (Basel). 2026 Apr 29;18(9):1426. doi: 10.3390/cancers18091426 (PMC13162811; doi:10.3390/cancers18091426)
Supplement: Supplementary file 1 [file cancers-18-01426-s001.zip › cancers-4244327-supplementary.pdf]

Supplementary Materials

Prognostic Impact of MYC/TP63 Molecular Subtypes in Adenoid Cystic Carcinoma: A Meta-analysis

Supplementary Sensitivity Analysis — 6-Cohort Model Including Economopoulou et al. (2025)

This supplementary material presents the results of a pre-planned sensitivity analysis incorporating the Economopoulou et al. (2025) cohort (ACC I n=8, ACC II n=39) into the primary meta-analysis. This cohort was excluded from the primary analysis because the ACC I subgroup contained fewer than 10 patients (predefined inclusion threshold). The 6-cohort analysis yielded a pooled hazard ratio (HR) of 3.84 (95% CI: 2.62–5.62), $I^2 = 0.0\%$, prediction interval 2.33–6.32, confirming robustness of the primary results.

Supplementary Figure S1: Forest Plot — 6-Cohort Sensitivity Analysis

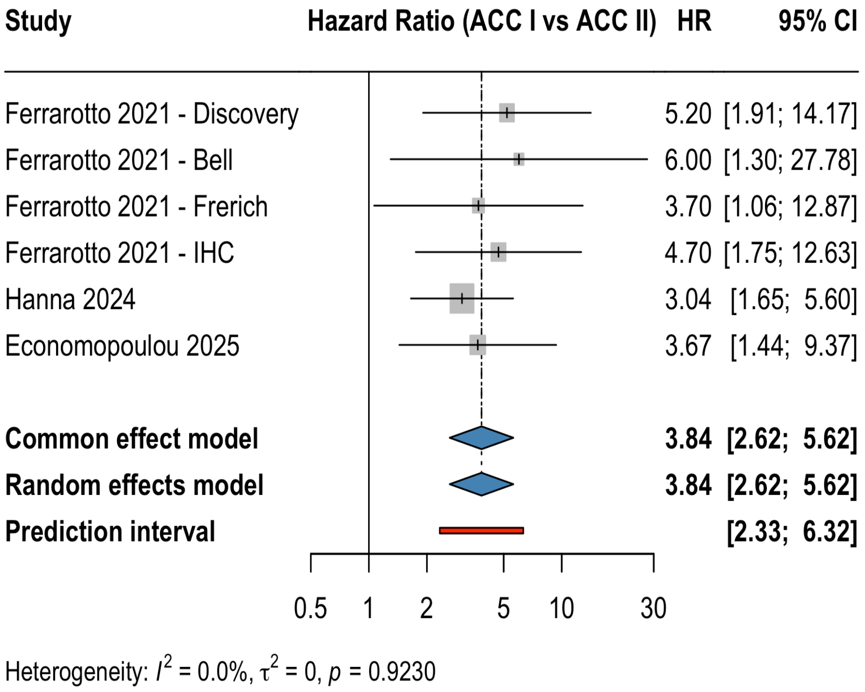

Forest plot of the supplementary sensitivity analysis including all six cohorts (Ferrarotto 2021 Discovery, Bell, Frerich, and IHC cohorts; Hanna 2024; Economopoulou 2025). Pooled HR = 3.84 (95% CI: 2.62–5.62), I^2 = 0.0%, prediction interval 2.33–6.32. Random-effects model (DerSimonian–Laird). Diamond represents the pooled estimate; horizontal bars represent 95% CI.

Supplementary Figure S2: Subgroup Analysis by Classification Method — 6-Cohort Analysis

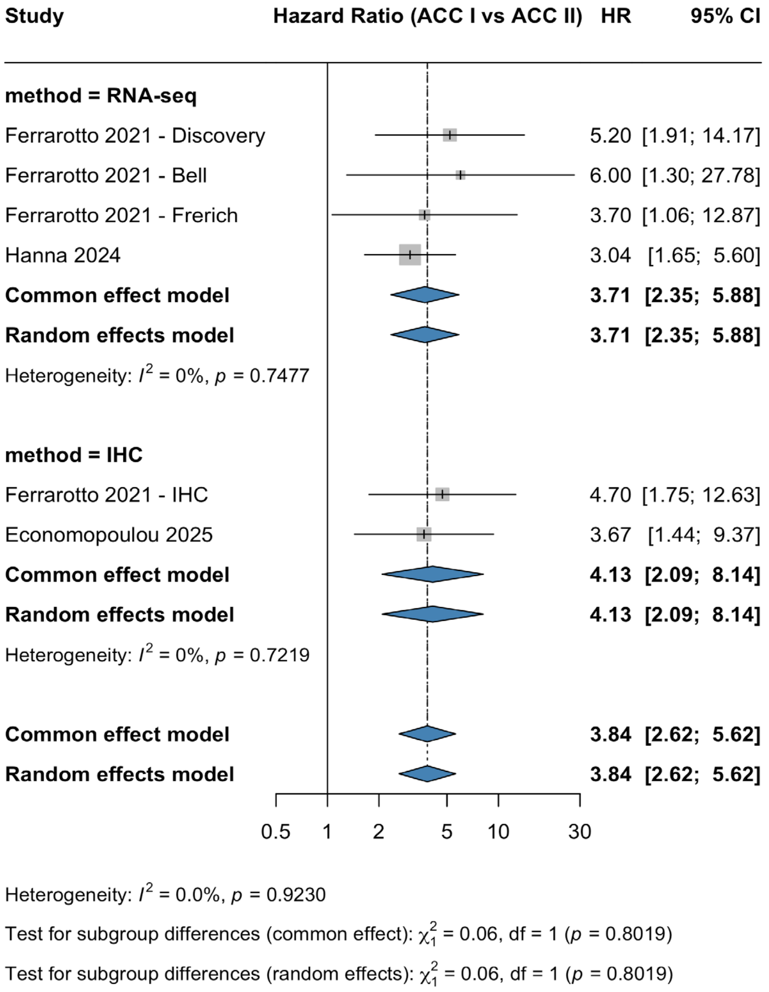

Subgroup forest plot stratified by molecular classification method (RNA sequencing vs. immunohistochemistry, IHC). RNA-seq subgroup (k=4): HR = 3.71 (95% CI: 2.35–5.88), $I^2 = 0\%$. IHC subgroup (k=2): HR = 4.13 (95% CI: 2.09–8.14). $\tau^2 = 0$ for both subgroups. No statistically significant subgroup difference detected (p = 0.8019).

Supplementary Figure S3: Leave-One-Out Sensitivity Analysis — 6-Cohort Analysis

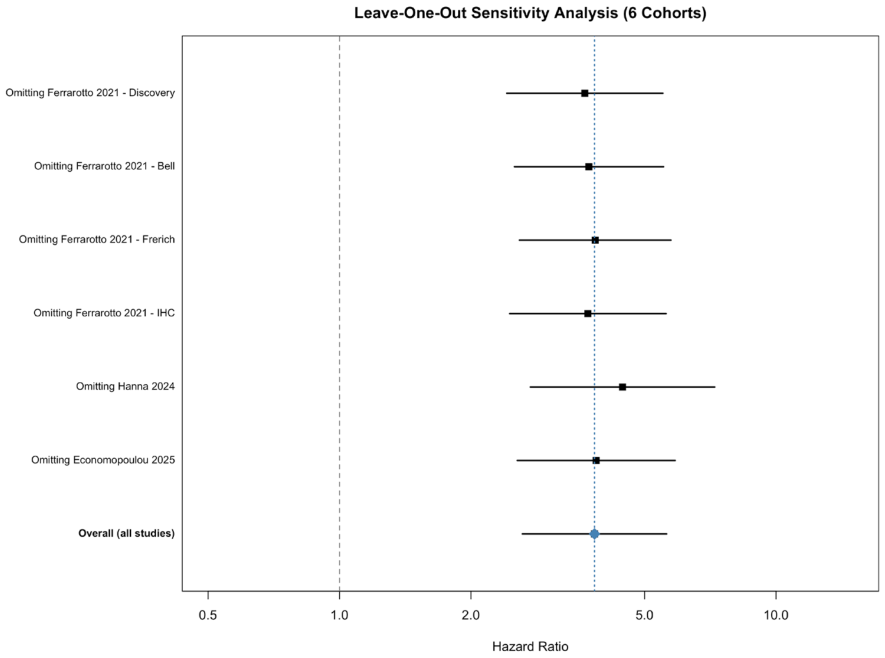

Leave-one-out sensitivity analysis for the 6-cohort model. Each row shows the pooled HR after systematically omitting one cohort. All estimates remained statistically significant (all $p < 0.001$), confirming robustness of the primary result. Blue circle denotes the overall pooled estimate (all six cohorts included).

Supplementary Figure S4: Cumulative Meta-Analysis — 6-Cohort Analysis

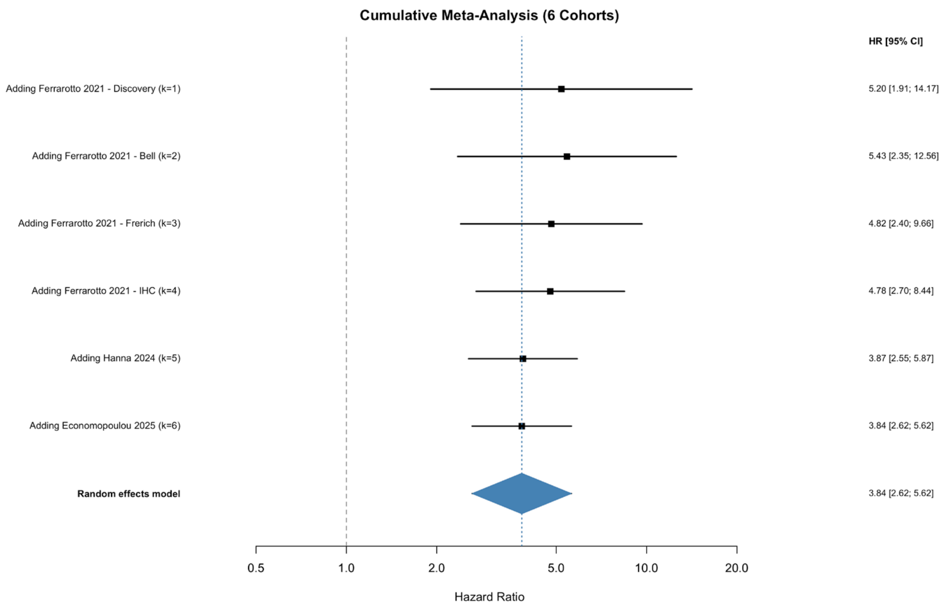

Cumulative meta-analysis showing the evolution of the pooled hazard ratio as cohorts are added sequentially in chronological order (Ferrarotto 2021 cohorts first, followed by Hanna 2024 and Economopoulou 2025). The estimate stabilised after the third cohort with progressive narrowing of confidence intervals. Blue diamond denotes the final pooled estimate.

Supplementary Figure S5: Influence Diagnostics — 6-Cohort Analysis (Exploratory)

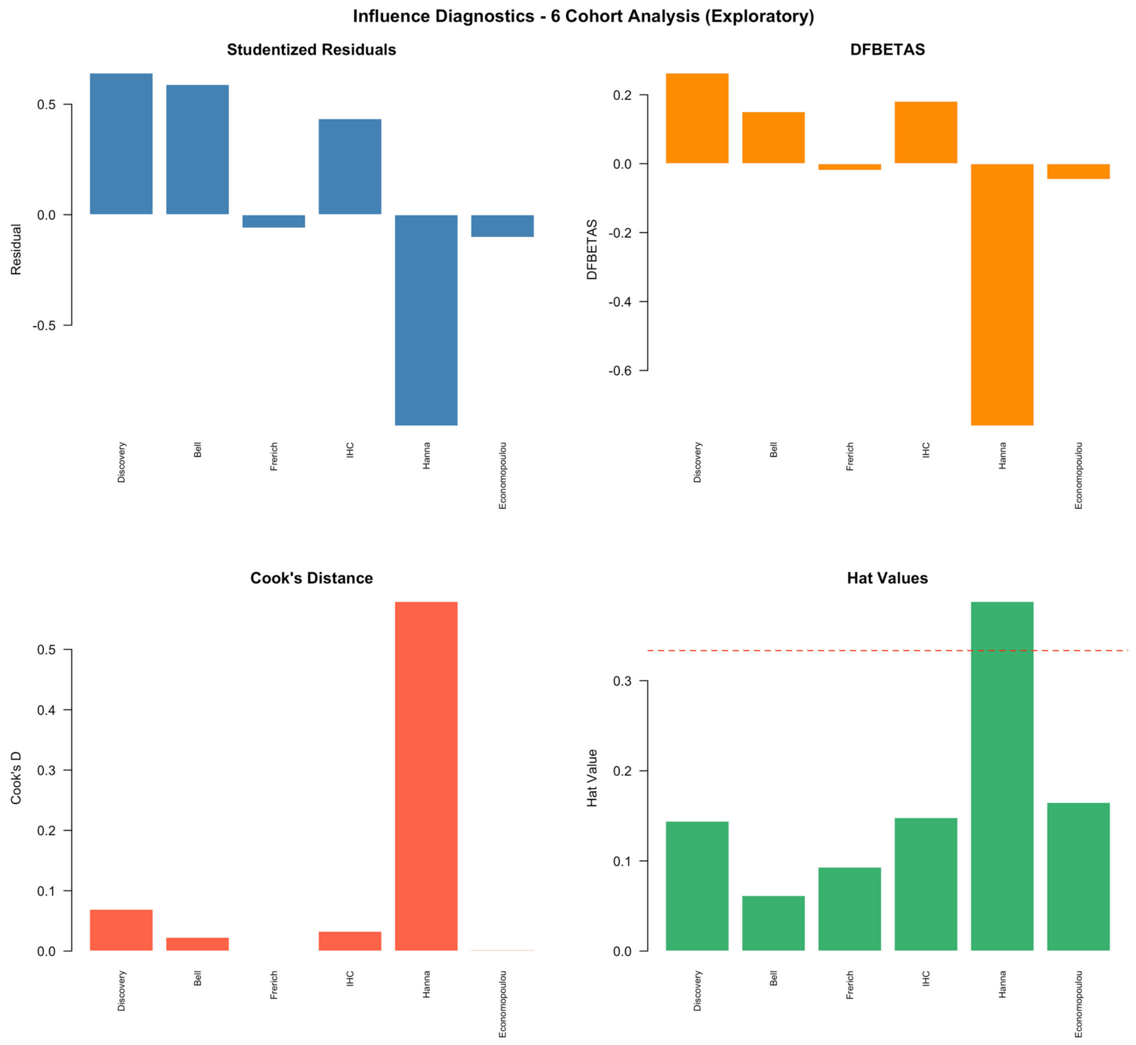

Four-panel influence diagnostic plot showing (top-left) studentized residuals, (top-right) DFBETAS, (bottom-left) Cook's distance, and (bottom-right) hat values (leverage). Dashed red lines indicate conventional thresholds. No cohort exceeded the Cook's distance threshold of 1.0. This is an exploratory analysis given the small number of cohorts (k=6).

Supplementary Figure S6: The PRISMA 2020 checklist

Section	Item	Checklist Item	Location
TITLE	1	Identify the report as a systematic review	Page 1
ABSTRACT	2	Structured abstract (PRISMA for abstracts)	Page 1–2
INTRODUCTION	3	Rationale	Page 2–3
INTRODUCTION	4	Objectives	Page 3
METHODS	5	Eligibility criteria	Page 4–5
METHODS	6	Information sources	Page 4
METHODS	7	Search strategy (summary provided)	Page 4
METHODS	8	Selection process (2 reviewers)	Page 4–5
METHODS	9	Data collection process	Page 5
METHODS	10a	Outcomes (OS defined)	Page 5
METHODS	10b	Other variables	Page 5
METHODS	11	Risk of bias (NOS, 2 reviewers)	Page 5–6
METHODS	12	Effect measures (HR)	Page 6
METHODS	13a	Eligibility for synthesis	Page 4–5
METHODS	13b	Data preparation (HR extraction/digitization)	Page 5
METHODS	13c	Data presentation (forest plots)	Page 6
METHODS	13d	Synthesis methods (random-effects DL)	Page 6
METHODS	13e	Heterogeneity exploration (subgroup/meta-regression)	Page 6
METHODS	13f	Sensitivity analysis (leave-one-out)	Page 6
METHODS	14	Reporting bias (funnel plot)	Page 6
METHODS	15	Certainty assessment (NOS-based quality)	Page 5–6
RESULTS	16a	Study selection + flow diagram	Page 7
RESULTS	16b	Excluded studies with reasons	Page 7
RESULTS	17	Study characteristics	Page 7–8
RESULTS	18	Risk of bias (NOS scores)	Page 8
RESULTS	19	Individual study results	Page 8–9
RESULTS	20a	Summary of contributing studies	Page 7–8
RESULTS	20b	Meta-analysis results	Page 8–9
RESULTS	20c	Heterogeneity exploration	Page 9
RESULTS	20d	Sensitivity analysis	Page 9–10
RESULTS	21	Reporting bias assessment	Page 11
RESULTS	22	Certainty of evidence (consistent NOS high quality)	Page 8
DISCUSSION	23a	Interpretation of results	Page 12
DISCUSSION	23b	Limitations of evidence	Page 13

Section	Item	Checklist Item	Location
DISCUSSION	23c	Limitations of review process	Page 13
DISCUSSION	23d	Implications for practice/research	Page 13–14
OTHER	24a	Registration (not registered)	Page 4
OTHER	24b	Protocol (not prepared)	Page 4
OTHER	24c	Amendments	Not applicable
OTHER	25	Support (no funding)	Page 14
OTHER	26	Competing interests	Page 14
OTHER	27	Data availability (derived from published literature)	Page 14
